# Treatment for benign skin lesion in zygomatic-infraorbital region by the expanded multi-lobe cervicofacial advancement rotation flap in pediatric patients

**DOI:** 10.1186/s12893-024-02312-7

**Published:** 2024-01-13

**Authors:** Yucheng Yan, Bing Han, Cheng Gan, Jincai Fan, Liqiang Liu, Lu Zhou, Jipeng Song

**Affiliations:** 1 Comprehensive Ward of Plastic Surgery Hospital, Chinese Academy of Medical Sciences and Peking Union Medical College, Beijing, China; 2 Scar and Wound Treatment Center, Plastic Surgery Hospital, Chinese Academy of Medical Sciences and Peking Union Medical College, Beijing, China

**Keywords:** Facial defect, Surgical flaps, Tissue expansion, Pediatrics

## Abstract

**Purpose:**

Benign skin lesions in zygomatic-infraorbital regions severely influence pediatric patients’ appearance as well as mental health. Treatments are difficult for the high requirements of patients’ guardians in both function and aesthetics. The present study aims to introduce a surgical method, Expanded Multi-Lobe Cervicofacial Flap, which combines the advantages of the classical cervicofacial advancement rotation flap and the tissue expansion technique.

**Methods:**

A total of 21 pediatric patients were enrolled. The treatment process included 2 stages: implantation of the skin tissue expander and flap transfer. The excessive skin created by tissue expansion extended the coverage area of the multi-lobe flap.

**Results:**

In this retrospective study, follow-up periods were all more than 12 months (20.8 ± 6.7). In the last follow-ups, the flaps were all in good condition, and No facial organ displacement was observed. The patients’ guardians were satisfied with the outcomes.

**Conclusions:**

Using the expanded multi-lobe cervicofacial flap for the zygomatic-infraorbital benign skin lesion repair is effective, and this method is especially applicable to the pediatric population.

## Introduction

Benign skin lesions in zygomatic-infraorbital regions are commonly encountered in the clinical practice of plastic surgery, and the affected patients usually come to clinic in childhood. The treatments are of great difficulty for the reason that only the complete lesion resection and good coverage of wound areas are far from enough. The aesthetic and functional reconstructions of zygomatic-infraorbital regions are closely related to surgical outcomes and patient satisfaction.

For the large lesions, greater than 2 cm, close to lower eyelids, the wide defects after resection cannot be sutured directly. The unmatched color and texture of the distant flap compromise the esthetic outcome. High technical requirements and the long surgical duration of free flap transfer limit its application. Local flaps have similar skin characteristics to the recipient area. Juri reported the Cervicofacial Advancement Rotation Flap for facial repairs [1], which is regarded as the classical repair treatment of medium-large facial defects [2]. However, to transfer a large amount of skin tissue, the incision would be extended to the lower neck and even the chest. For pediatric patients with tight skin, the amount of local transferable tissue is relatively less. To compensate for the deficiency, a larger area of dissection is needed which would increase the surgery difficulty and result in more serious scars.

Since Neumann introduced the technique of soft tissue expansion in 1957 [3], it has become an indispensable tool in the reparative and reconstructive surgery fields. This technique could generate extra skin tissue having similar thickness, color, texture even sensibility to the adjacent skin, which just meets the high aesthetic demands in facial reconstructions [4]. However, the post-expansion contraction would affect the long-term result and cause disfigured complications [5]. An unreasonable advancement method of expanded skin flap will induce obvious facial scars.

In our clinical practice, we found that using the excessive skin tissue created by soft tissue expansion could reduce the surgical trauma caused by the classical cervicofacial advancement rotation flap. On the other hand, the technique of the advancement rotation flap allows the redistribution of facial skin tissue, which helps to solve the problem of post-expansion contraction. Therefore, we led this retrospective study and performed long-term follow-up to share our experience in treating the benign skin lesion in the zygomatic-infraorbital region.

## Methods

We conducted a retrospective study that covered the patients with benign skin lesions in zygomatic-infraorbital regions who underwent the surgery of expanded multi-lobe cervicofacial advancement rotation flap by the same surgery team from 2018.1 to 2022.3. The inclusion criteria were listed as follows: (1) Age less than 18 years old; (2) the benign lesion was confirmed by the postoperative pathological examination or trauma history; (3) no factor affecting blood supply of normal facial skin, such as the history of facial radiotherapy and surgery; (4) willing to cooperate with long-term treatments. The present research was approved by Institutional Review Board (IRB)/ Ethics Committee of Plastic Surgery Hospital, Chinese Academy of Medical Sciences and Peking Union Medical College. Informed consent were obtained from all the patients/ patients’ guardians, for both study participation and publication of identifying information/images.

### Surgical technique

The surgical procedure was divided into two stages: implantation of the tissue expander and rotation advancement of the expanded cervicofacial flap. All procedures were performed with the same treatment team.

### Stage 1

**Fig. 1:**
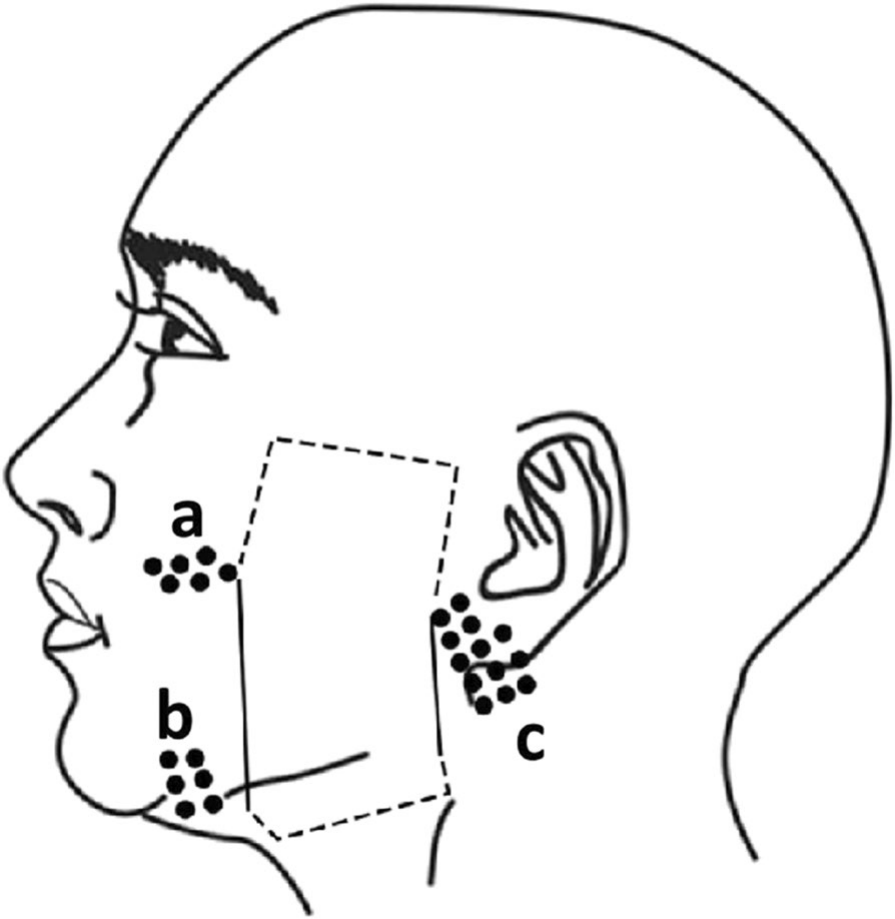
**A** The schematic diagram of the implant placement. **a**, zygomatic bone; **b**, stylomandibular ligament; **c**,earlobe

A. Postoperative Design: Zygomatic bone, stylomandibular ligament, and earlobe were set as the bound. The inferior and superior surgical boundary varied depending on the size of the expander to be implanted, and the surgical area of stage 1 could be downward extended to the neck (Fig. 1A). As for the expander size, the rectangular expander whose base area was similar to the defect was chosen. Normally, the expander with a volume of 30-150 ml was implanted. The ideal water injection volume would expand the expander 2–3 times its original size.
B. Surgical Technique: The incision was made around the earlobe of the affected side. Dissection was performed adhering to the bottom surface of the facial subcutaneous adipose tissue and avoided undermining the superficial musculo-aponeurotic system (SMAS). Preserved facial artery perforators near the boundary of the dissection region and placed the expander after good hemostasis. Then the incision was sutured with reduced-tension.

### Stage 2

**Fig. 2:**
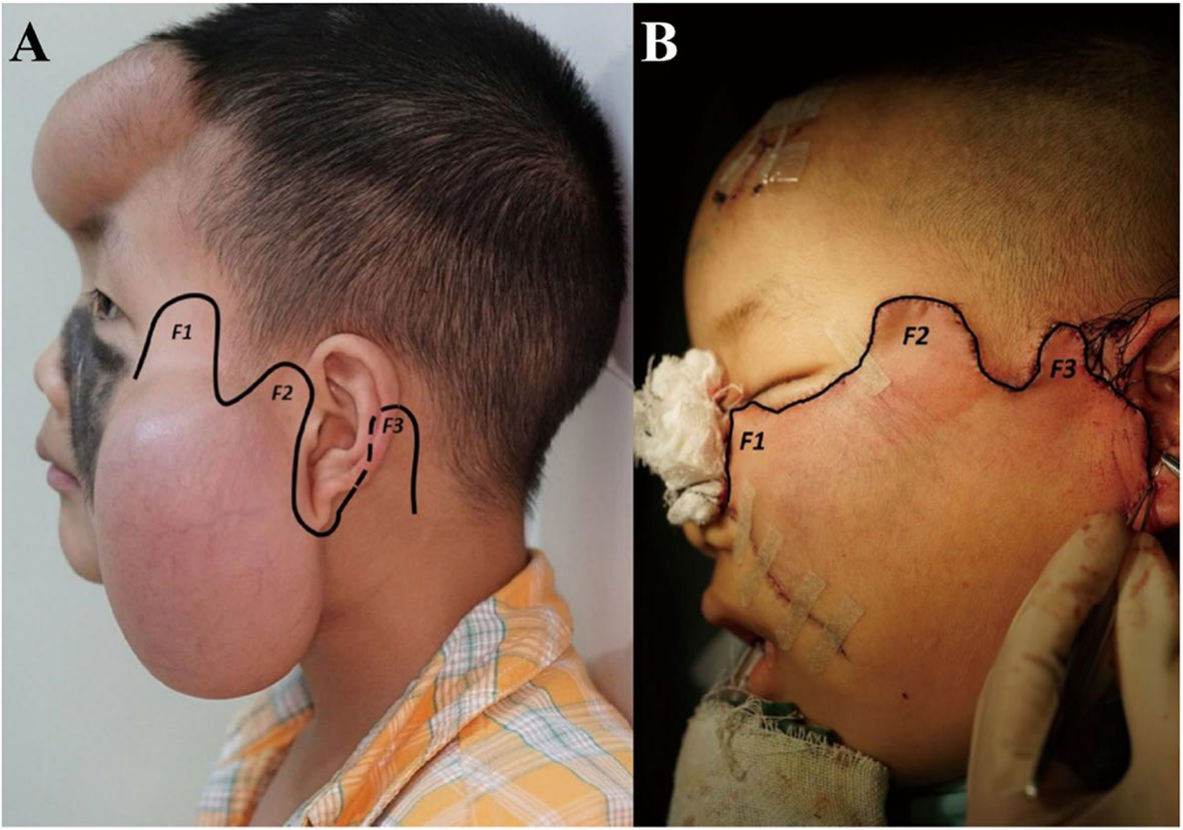
**A** The schematic shows the three lobes of the cervicofacial advancement rotation flap before surgery. **B** The immediate postoperative positions of the muti-lobe flap after advancement rotation. (F1: temporal skin flap; F2: preauricular skin flap; F3: postauricular skin flap)

The incision was designed along the hairline, ear root, the edge of the lesion, and nasolabial groove, which were all in the subunit borders of the cheek. Based on the design principle above, the facial skin around the lesion could be divided into three lobes: temporal skin flap (F1), preauricular skin flap (F2), and postauricular skin flap (F3). The size and shape of F1 were decided by the lesion to be excised, and the areas of F1–3 were designed successively decreased (Fig. 2A&B).

After tumescent local anesthesia, the skin was incised along the marked lines, and dissection was performed between SMAS and the subcutaneous fat layer. Subsequently, the lesion was excised completely, and the expander was removed to form the final multi-lobe cervicofacial flap. Similarly, the facial artery perforators were preserved for better blood supply.

**Fig. 3:**
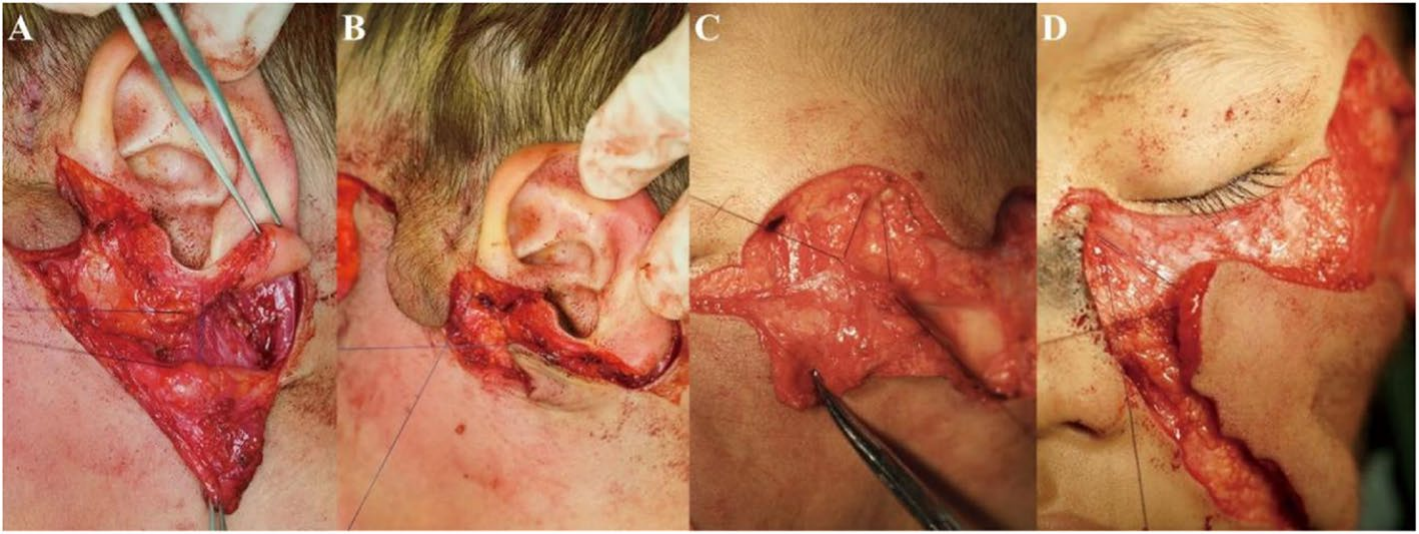
Four fixed structures of the flap. **A** platysma-auricular ligament; **B** periosteum of zygomatic arch; **C** deep temporal fascia; **D** medial canthal ligament

The advancement rotation of each lobe was performed sequentially: After forming F1–3, part of the expanded skin tissue was extended along the direction of the mandibular border to repair the postauricular defect left by F3. The platysma-auricular ligament was used to anchor the postauricular flap (Fig. 3A). Then, the other expanded skin tissue was extended to prolong the lengths of F2 and F3. At the same time, rotate the whole muti-lobe flap toward the facial midline. F3 was used to cover the preauricular wound left by F2 and was fixed to the periosteum of the zygomatic arch (Fig. 3B). F2 was transferred to repair the temporal wound caused by forming F1, and the deep temporal fascia serves to anchor F2 (Fig. 3C). Finally, F1 was transferred to the zygomatic-infraorbital region. The distal end of F1 was fixed on the medial canthal ligament (Fig. 3D). Finally, careful closure with tight sutures in layers was performed (Fig. 2B). All patients received dexamethasone 5 mg i.v. immediately after surgery and twice daily in two postoperative days to prevent edema and ischemia-reperfusion injury [6].

## Results

A total of 21 patients with benign skin lesions in zygomatic-infraorbital regions were enrolled in the research, and their preoperative information was summarized in Table 1. Among them, 16 patients had giant melanocytic nevus while 2 patients with verrucous epidermal nevus, 2 patients with verrucous hemangiomas, and 1 patient with burn scar. The age ranged from 3 to 16 years old, with an average of 6.1 ± 3.0. The postoperative follow-up period of all patients enrolled was more than 12 months, and the average duration was 20.8 ± 6.7 months.

**Table 1:**
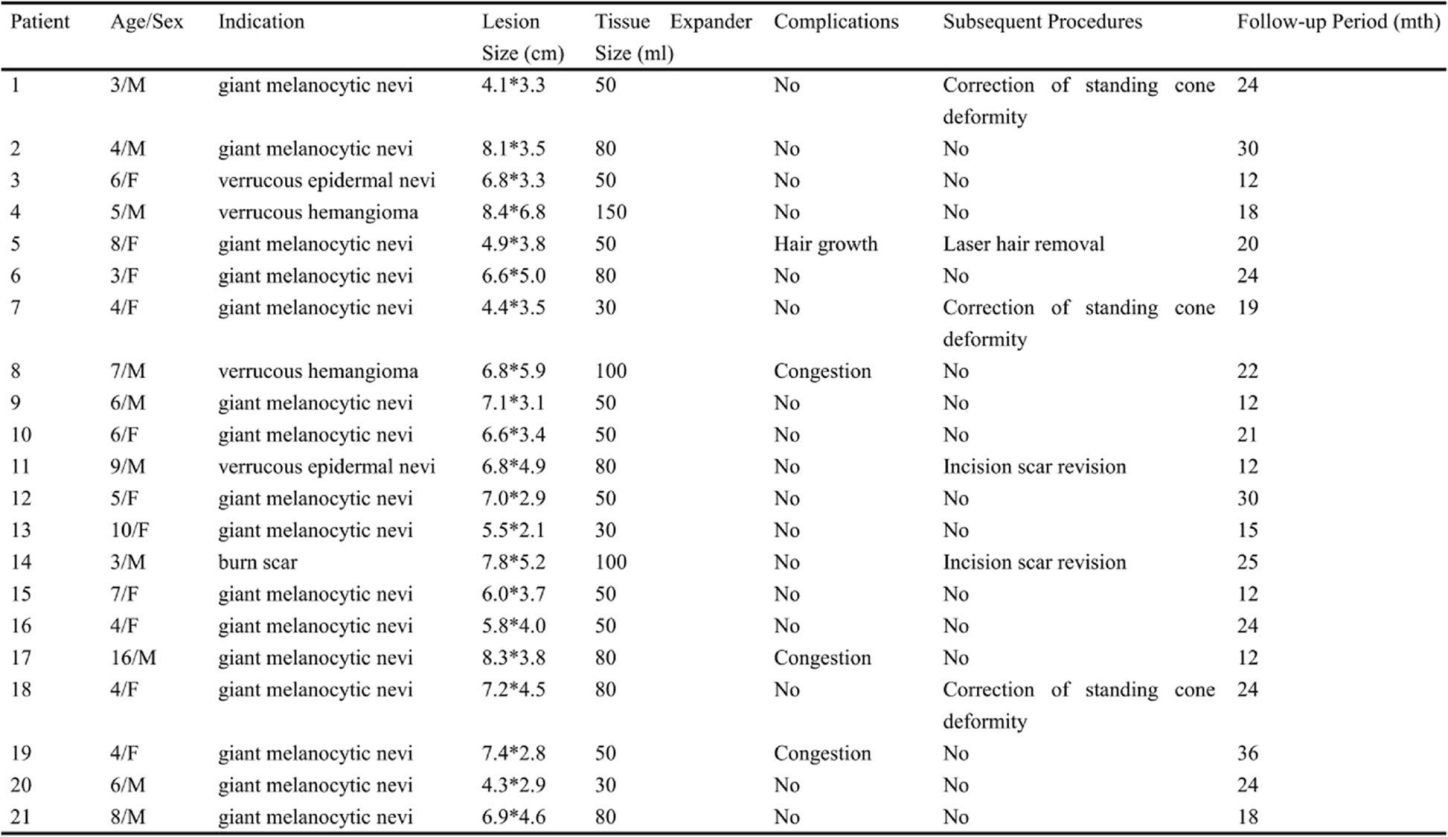
Information of the patients enrolled

All the Multi-Lobe Cervicofacial Advancement Rotation Flaps survived. Among the 21 flaps, 3 of them had venous congestion in distal ends, and were resolved after positive drainage. Hair was observed on the flap in 1 patient. 3 patients were observed with standing cone deformities in check, and performed correction surgeries. 2 patients sought revision surgeries because of the scar left by the primary surgeries. No patients were observed with flap bulk, flap ischemia, eyelid ectropion, and other complications. All the patients/ patients’ guardians were satisfied with the treatment outcomes in the final follow-ups.

### Typical cases

#### Case 1

**Fig. 4:**
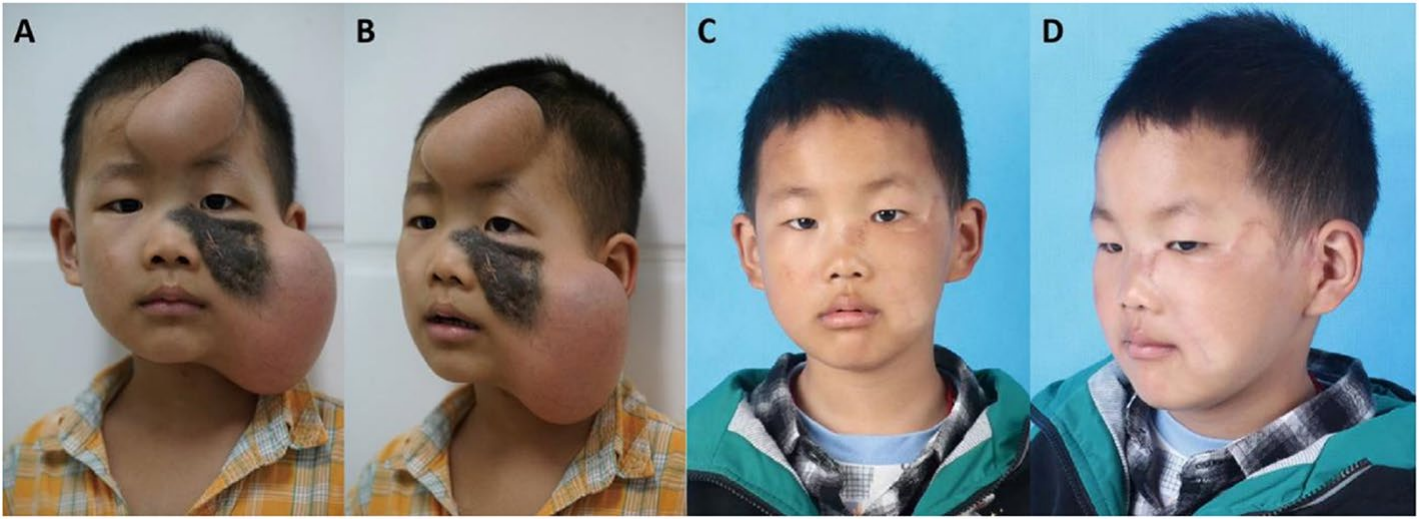
**A **& **B** the photographs of case 1 with two sufficient-expanded skin tissue expanders before the stage 2 surgery. **C **& **D** 30 months after the stage 2 surgery. The expanded multi-lobe cervicofacial advancement rotation flap presented with good color, texture, and thickness.

A 4-year-old boy with a giant melanocytic in his left zygomatic-infraorbital region sought treatments at our hospital. The lesion was measured at 8.1 × 3.5 cm, and an 80 ml skin expander was implanted to repair the zygomatic-infraorbital region. Meanwhile, the excessive skin tissue created by a 30 ml skin expander in the fontal region was used for skin transplantation to repair the wound on the nose dorsum. The follow-up period was 30 months, and the patient’s guardians were quite satisfied with the outcome of the final follow-up (Fig. 4). Using tension-reducing tapes and silicone gel dressings caused the lightness of skin color in the left temporal and check regions.

#### Case2

**Fig. 5:**
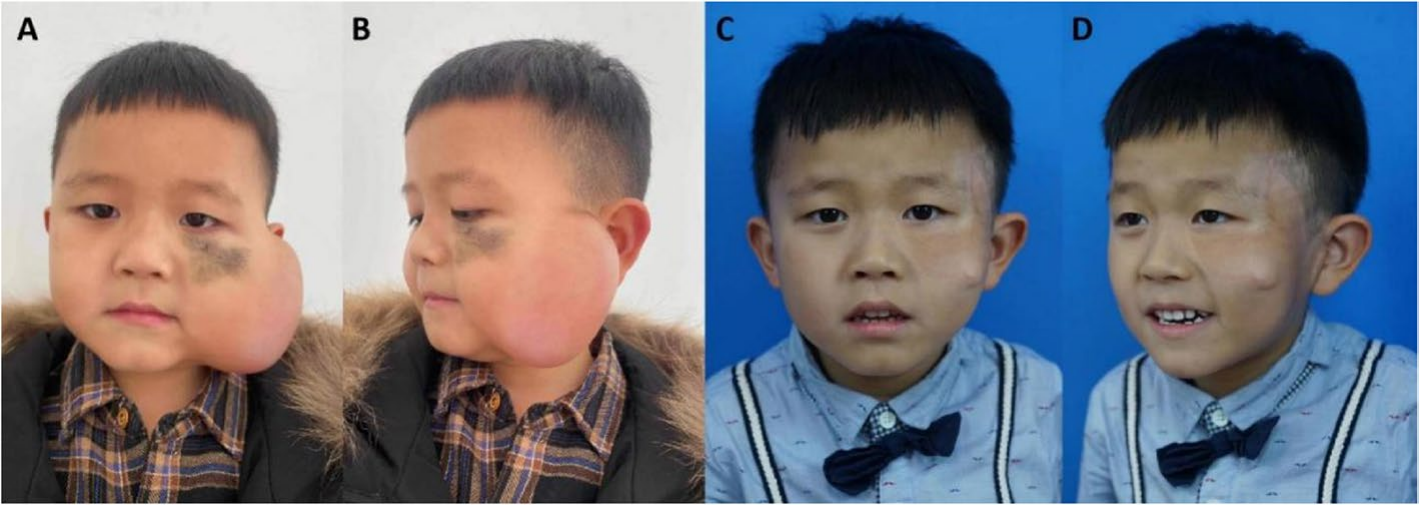
**A **& **B** the photographs of case 2 with one sufficient-expanded skin tissue expander. **C **& **D** 24 months after the surgery of the expanded multi-lobe cervicofacial advancement rotation flap. The flap presented with good color, texture and thickness. The sequential surgery was performed to correct the standing cone deformity

A 3-year-old boy had a giant melanocytic in his left zygomatic-infraorbital region measured 4.1*3.3 cm. A 50 ml skin expander was implanted for the following surgeries. After regular water injection, the surgery of the expanded multi-lobe cervicofacial advancement rotation flap was performed, and the facial lesion was totally excised. 24 months later, the flap was in good condition, and the patient’s guardians were very satisfied with the surgical outcome. Using tension-reducing tapes and silicone gel dressings caused the lightness of skin color in the left temporal region. The patient received sequential surgery for standing cone deformity correction (Fig. 5).

## Discussion

The common species of benign skin lesions included giant melanocytic nevi, verrucous epidermal nevi, verrucous hemangioma, burn scars, and so on [7]. The primary harm of them is impairing physical appearance not life-threatening. For young patients effective treatments are crucial to their mental health and personal development, and ought to be paid enough attention.

Repairment of zygomatic and infraorbital regions is challenging because of the proximity of the nose and lower eyelid [8, 9]. The poorly designed flap could cause significant organ displacement, which would aggravate the patient’s suffering. After years of clinical practice, the cervicofacial flap (CF flap) was verified as a reliable surgical method for repairing large facial defects [2]. CF flap utilizes the extensibility and laxity of facial, cervical, even chest skin to form the local flap, which guarantees the similarity in texture and color [10]. What’s more, the incisions along the borders of the facial subunits are relatively not obvious. However, middle-aged and elderly people with loosened skin tissue are more suitable for this procedure [11, 12]. In view of that most patients with facial benign skin tumors seek treatment at a young age, the scar spanned face, neck, and chest will inevitably impair the appearance, neck motion, and child development, especially for the Asian population with a higher tendency in scar formation and hyper-pigmentation [13].

**Fig. 6:**
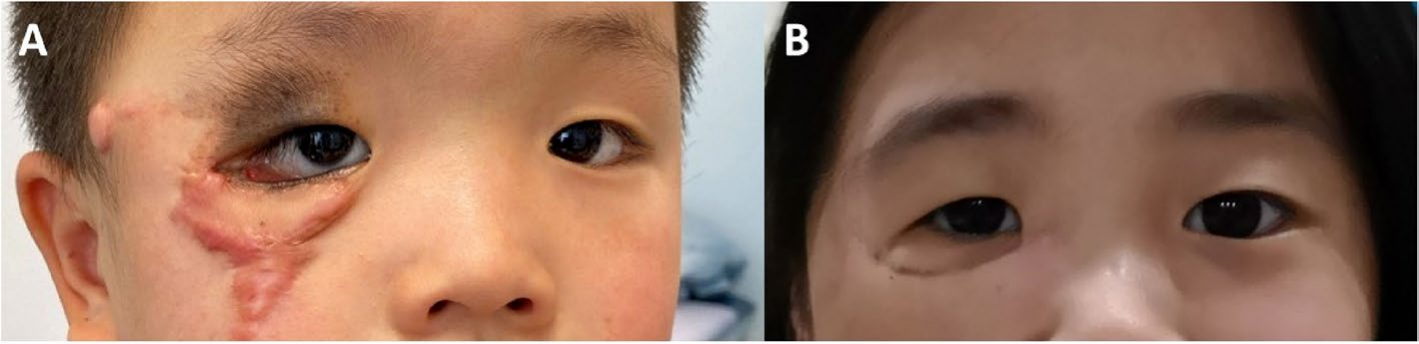
**A** Obvious scar and lower eyelid ectropion caused by poorly designed expanded flap transfer. **B** Obvious asymmetry resulted from contraction of the expanded flap

It is widely acknowledged that the technique of tissue expansion has many strengths: harvesting a larger flap with less donor site injury; enhancing vascularization; and the expanded flap has similar color, texture, and thickness to the recipient site [14]. Besides the facial skin tissue, the cervical region is also an ideal donor site for tissue expansion in repairing facial defects [15]. However, the postoperative contraction of the poorly designed expanded flap significantly affected the long-term treatment result. In the zygomatic-infraorbital region, lower eyelid deformations and obvious scars are common complications in clinical practice (Fig. 6).

In view of the complementarity of the two techniques above, the idea of enlarging the CF flap with a soft tissue expander was generated. With the guidance of previous studies, the method of expanded multi-lobe cervicofacial advancement rotation flap was successfully used in zygomatic-infraorbital regions of pediatric patients.

1. The benign nature of the lesion allows staged procedures to achieve a better treatment result. For pediatric patients, according to our clinical experience, normally ones over 3 years old could cooperate with water injections of expanders, which would last for half a year. Thus, to guarantee pediatric patients’ socialization and psychological development, we suggest that the patients over 3 years old should receive treatments as early as possible.
2. The soft tissue expander was placed above the mandible. The deep osseous structure led to a higher expansion efficiency for the reason that the height above the skin surface was greater. As for the bone deformity after tissue expansion, the bone changes would alleviate and recover gradually after removing the expander [16]. What’s more, the implantation region is far away from facial organs, and they wouldn’t be overstretched during the long period of tissue expansion. In this method of expander implantation, the maxilla, the zygomatic bone, and the zygomatic arch would be protected to the maximum extent. Only in the late phase of tissue expansion, the osseous structures mentioned above would be compressed.
3. The facial artery goes under the level of SMAS, and the facial subcutaneous vascular communication is complex. The above reasons hinder the formation of facial axial skin flaps [10]. However, the well-perfused facial skin makes the necrosis rate of CF flap at a low rate [8]. Combining the technique of soft tissue expansion, which enriches the blood supply more effectively than the delay phenomenon [4], the necrosis rate would be even lower in theory. In the present study, all flaps survived and no partial necrosis was recorded. When the nasal dorsum was affected by benign lesions, considering the different facial subunits and avoiding excessive pulling of the flap, the full sickness skin grafting or skin flap transfer was performed after excising the lesion on the nasal dorsum. In this way, postoperative appearance would be more natural and the ischemic risk would also be reduced.
4. Supporting suture is of great significance for preventing the disfiguring postoperative contracture. To avoid the ectropion of the lower eyelid, F1 was fixed on the periosteum and F2 was fixed on the deep temporal fascia. Both deep anatomical structures are compact and tough enough for fixation [17]. These two eyelid medial and lateral supporting sutures could maintain the figure of the eyelid effectively.
5. The rotation center was located in the lateral cheek. When it was close to the mandible, the standing cone deformity could be relieved by lifting the excessive skin tissue along the direction of the mandibular border to repair the postauricular defect. What’s more, the mild standing cone deformity in the lower later check is similar to the contour of the contralateral buccal fat pad in young patients. If the standing cone deformity is still obvious after compression therapy, it could be treated with a selective surgery.

The main limitation of this surgical method is the relatively high requirement for surgeons in surgery skills and clinical experience. Though the skin dissection area of the present method was much smaller than the classical CF flap, the dissection of the facial lesion was still challenging and time-consuming. Meticulous manipulation would avoid the risk of injuring the great auricular nerve and facial nerve. The well-preserved facial artery perforators would provide a better blood supply for the multi-lobe flaps. What’s more, because of the complexity of zygomatic-infraorbital soft tissue and individual differences, whether using the technique of tissue expansion needs specific judgment. If the expanded flap is necessary, we recommend this method. Further studies, like digitalization techniques, are needed to provide exact indications and simplify the postoperative designation.

## Conclusion

The method of expanded multi-lobe cervicofacial advancement rotation flap combines the advantages of the classical cervicofacial advancement rotation flap and the tissue expansion technique, and meanwhile minimizes postoperative complications and surgical trauma. In the present study, long-term follow-up proved that this method is an optimal choice for repairing benign skin lesions in the zygomatic-infraorbital region, especially for the young patients with high aesthetic requirements.

## Data Availability

The authors confirmed that the data supporting the findings of this study are available within the article.

## References

[1] Juri J, Juri C (1979). Advancement and rotation of a large cervicofacial flap for cheek repairs. Plast Reconstr Surg..

[2] Cass ND, Terella AM (2019). Reconstruction of the cheek. Facial Plast Surg Clin North Am..

[3] Neumann CG (1946). (1957) the expansion of an area of skin by progressive distention of a subcutaneous balloon; use of the method for securing skin for subtotal reconstruction of the ear. Plast Reconstr Surg..

[4] Wang C, Yang S, Zhang J, Yan L, Song P, Hyakusoku H, Pu LLQ (2017). An overview of pre-expanded perforator flaps. Clin Plast Surg..

[5] Van Damme PA, Heidbuchel KL, Kuijpers-Jagtman AM, Maltha JC, Freihofer HP (1992). Cranio-maxillo-facial tissue expansion, experimentally based or clinically empiric? A review of the literature. J Craniomaxillofac Surg..

[6] Corrick RM, Tu H, Zhang D, Barksdale AN, Muelleman RL, Wadman MC, Li YL (2018). Dexamethasone protects against tourniquet-induced acute ischemia-reperfusion injury in mouse hindlimb. Front Physiol..

[7] Salavastru CM, Butacu AI, Fritz K, Eren S, Tiplica GS (2022). Benign skin neoplasms in children. Hautarzt..

[8] Sakellariou A, Salama A (2014). The use of cervicofacial flap in maxillofacial reconstruction. Oral Maxillofac Surg Clin North Am..

[9] Roth DA, Longaker MT, Zide BM (1998). Cheek surface reconstruction: best choices according to zones. Oper Tech Plast Reconstr Surg..

[10] Chen S, Li Y, Yang Z, Ma N, Wang W, Xu L, Liu Q (2019). Surgical treatment for facial port wine stain by prefabricated expanded cervical flap carried by superficial temporal artery. J Craniofac Surg..

[11] Seretis K, Boptsi A, Boptsi E, Lykoudis EG. The ‘facelift’ flap revisited. J Craniofac Surg Publish Ahead of Print. 2022; 10.1097/SCS.0000000000009082. 10.1097/SCS.0000000000009082 36730886

[12] Eroglu L, Simsek T, Gumus M, Aydogdu IO, Kurt A, Yildirim K (2013). Simultaneous cheek and lower eyelid reconstruction with combinations of local flaps. J Craniofac Surg..

[13] Kim S, Choi TH, Liu W, Ogawa R, Suh JS, Mustoe TA (2013). Update on scar management: guidelines for treating asian patients. Plast Reconstr Surg..

[14] Ding J, Li Y, Li W, Liu C, Liu H, Cui J, Su Y, Ma X (2018). Use of expanded deltopectoral skin flaps for facial reconstruction after sizeable benign tumor resections. Am J Transl Res..

[15] Gao B, Xiao K, Zhu H, Sheng L, Yu Q, Mao X, Li Q, Xie F (2016). An algorithm for using expanded cervical flaps to resurface facial defects based on five different methods. Burns..

[16] Qiu Y, Qiao C, Yang J, Jin Y, Chen H, Lin X (2019). Forehead deformities after tissue expansion: retrospective analysis and recommendations. J Plast Reconstr Aesthet Surg..

[17] Akita K, Fukino K (2022). The significance and classification of the layered structures of the human masseter and temporalis. Ann Anat..

